# Investigating Molecular Determinants of Cancer Cell Resistance to Ionizing Radiation Through an Integrative Bioinformatics Approach

**DOI:** 10.3389/fcell.2021.620248

**Published:** 2021-04-07

**Authors:** Halil Ibrahim Toy, Gökhan Karakülah, Panagiota I. Kontou, Hani Alotaibi, Alexandros G. Georgakilas, Athanasia Pavlopoulou

**Affiliations:** ^1^Izmir Biomedicine and Genome Center, Izmir, Turkey; ^2^Izmir International Biomedicine and Genome Institute, Dokuz Eylül University, Izmir, Turkey; ^3^Department of Computer Science and Biomedical Informatics, University of Thessaly, Lamia, Greece; ^4^DNA Damage Laboratory, Department of Physics, School of Applied Mathematical and Physical Sciences, Zografou, National Technical University of Athens, Athens, Greece

**Keywords:** ionizing radiation, DNA damage repair, cancer cell radioresistance, bioinformatics, gene expression profiles, biomarkers

## Abstract

Eradication of cancer cells through exposure to high doses of ionizing radiation (IR) is a widely used therapeutic strategy in the clinical setting. However, in many cases, cancer cells can develop remarkable resistance to radiation. Radioresistance represents a prominent obstacle in the effective treatment of cancer. Therefore, elucidation of the molecular mechanisms and pathways related to radioresistance in cancer cells is of paramount importance. In the present study, an integrative bioinformatics approach was applied to three publicly available RNA sequencing and microarray transcriptome datasets of human cancer cells of different tissue origins treated with ionizing radiation. These data were investigated in order to identify genes with a significantly altered expression between radioresistant and corresponding radiosensitive cancer cells. Through rigorous statistical and biological analyses, 36 genes were identified as potential biomarkers of radioresistance. These genes, which are primarily implicated in DNA damage repair, oxidative stress, cell pro-survival, and apoptotic pathways, could serve as potential diagnostic/prognostic markers cancer cell resistance to radiation treatment, as well as for therapy outcome and cancer patient survival. In addition, our findings could be potentially utilized in the laboratory and clinical setting for enhancing cancer cell susceptibility to radiation therapy protocols.

## Introduction

Radiation therapy or radiotherapy (RT) represents one of the optimal, most widely used modalities in the treatment of multiple cancers, either alone or combined with other curative anti-cancer modalities like chemotherapy (Delaney et al., 2005; Begg et al., 2011) or immunotherapy (Tang et al., 2014; Schoenhals et al., 2016). It is estimated that approximately 50% of all cancer patients worldwide undergo radiotherapy throughout their illness trajectory (Baskar et al., 2012).

Advances in radiotherapy contribute greatly to cancer patients' improvement of overall survival and quality of life (Baskar et al., 2012). The aim of radiotherapeutic regimens is to specifically and efficiently sensitize cancer cells to IR in order to eliminate them and prevent cancer recurrence and relapse, minimizing at the same time the adverse effects of radiation on healthy tissue. RT affects cancer cells either directly, by inducing genomic (DNA) lesions, or indirectly, through the generation of DNA damaging intermediates through the interaction with water, like reactive oxygen/nitrogen species (ROS/RNS) and free radicals (e.g., hydrogen ion, hydroxide, etc.) (Mikkelsen and Wardman, 2003; Yamamori et al., 2012).

However, cancer cells have the capacity to develop incredible tolerability and resistance to RT, thereby evading death. Radioresistance represents a major limiting factor in the effective treatment of different types of cancers. The response of tumor cells to radiation depends both on the resistance mechanisms of the cells and also on the accelerated repopulation of the tumor bulk by cells that have developed further radioresistance (Pavlopoulou et al., 2016, 2017). As noted in previous studies, the genes that are differentially expressed (either up- or down-regulated) between radioresistant (RR) and radiosensitive (RR) cancer cells are generally implicated in DNA damage response and repair (DDR/R) pathways, apoptosis, hypoxia, or response to oxidative stress, etc. (Pavlopoulou et al., 2016, 2017). The complexity of radiation resistance mechanisms suggests the involvement of different and diverse biological mechanisms.

During the last decade, the advances in high-throughput (HTP) “omics” technologies (e.g., RNA-Seq and microarrays) enabled the generation of an enormous amount of gene expression data. Data produced with HTP technologies are stored in international public repositories such as NCBI's GEO (Gene Expression Omnibus) (https://www.ncbi.nlm.nih.gov/gds/) (Barrett et al., 2013; Clough and Barrett, 2016). GEO DataSets contains both original records and curated datasets.

Accumulated knowledge over years of research on the biological effects of radiation points toward the development of holistic approaches to “big data” analysis by employing systems biology methodologies (Unger, 2014; Beheshti et al., 2019; Spratt and Speers, 2019; Kanakoglou et al., 2020). Herein, we employed a rigorous systems biology approach to unravel the molecular determinants of resistance of cancer cells to IR, based solely on HTP data. To this end, publicly available transcriptome datasets relevant to cancer cell response to radiation were retrieved from GEO, and specifically, cancer cell lines that displayed enhanced resistance to radiation. Statistical analyses were carried out to identify the differentially expressed genes (DEGs) between radioresistant and radiosensitive tumor cells. Furthermore, functional annotation of genes allowed us to identify specific biological pathways implicated in cancer cell resistance to radiation. Our findings could be applied in the laboratory and clinical setting as biomarkers for the design of targeted and personalized radiotherapy regimens in order to effectively sensitize cancer cells to radiation, enhance tumor control and thereby minimize tumor recurrence and metastasis.

## Methods

### Data Retrieval

The public repository NCBI GEO DataSets was searched extensively for gene expression datasets using relevant keywords: (“radiation therapy” or “radiotherapy”) and (“cancer” or “tumor”) and (“resistance” or “tolerability”) and (“sensitivity” or “responsive”) and (“human” or “homo sapiens”). A total of three eligible datasets were selected:

The GEO series GSE97543 (Emons et al., 2017) (Supplementary Table 1) includes global gene expression by microarray of both wild-type and radioresistant Dukes' type C colorectal adenocarcinoma (COAD) cell lines that were either non-irradiated or irradiated repeatedly with 2 Gray (Gy) of X-rays in order to acquire a radioresistant phenotype. The Agilent-026652 whole human genome microarray 4x44K, GPL13497 platform was used.

In GSE13280 (Marston et al., 2009) (Supplementary Table 1), genome-wide gene expression by microarray was performed of cell lines derived from pediatric B-precursor acute lymphoblastic leukemia (ALL) after 8 h *in vitro* exposure to 5 Gy IR. This dataset contains cell lines both resistant and responsive to radiation. The development of resistance and responsiveness to IR was assessed by measuring apoptosis in cells. The Affymetrix human genome U133A array, GPL96 platform was employed.

In GSE120798 (Gray et al., 2019) (Supplementary Table 1), three novel radioresistant breast cancer cell lines were established by exposing the corresponding parental cell lines MDA-MB-231 (metastatic mammary adenocarcinoma), MCF-7 (breast adenocarcinoma), and ZR-751 (luminal breast cancer) to increasing doses of X-rays for 2 and 8 h. Genome-wide gene expression analysis of both the parental and radioresistant cell lines (i.e., MDA/MDAR, MCF/MCFR, ZR/ZRR) was performed using high throughput sequencing. The NextSeq 550 (Homo sapiens), GPL21697 platform was used.

### Microarray-Based Transcriptomic Data Analysis

For each microarray study, the gene expression data that represent the gene expression summary for every probe and every sample were recorded. In microarrays, many probes can map to the same Gene Symbol for various reasons, and, conversely, a probe may also map to more than one Gene Symbol if the probe sequence is not specific enough. A simple approach would be to use only the probes with one-to-one mapping for further analysis; however, this approach results in the loss of important information. To conduct an analysis based on genes and not probes, the probe identifiers were firstly converted into gene identifiers, according to Ramasamy et al. (2008) guidelines. To this end, GPL files that contained information about the gene symbols that correspond to probe IDs were used in order to resolve the “many-to-many” relationships between probes and genes by averaging the expression profiles for genes with more than one probe (Ramasamy et al., 2008). We identified the Gene Symbols with the usage of the HUGO Gene Nomenclature Committee (Braschi et al., 2019) and the National Center for Biotechnology Information (NCBI) GENE (Sayers et al., 2020).

The two-sample *t*-test was employed to identify genes differentially expressed between the case (RR) and control (RS) groups. However, a disadvantage of the *t*-test in the analysis of microarray data is that if most of the experiments in a given study contain a relatively small number of samples per group, the assumption of normality is untenable. To resolve this, the statistical method *t*-test with bootstrap was used (Efron and Tibshirani, 1993). Bootstrap provides an ideal method to generate accurate estimates of the standard errors when no formula for the sampling distribution is available or when available formulas make inappropriate assumptions (e.g., small sample size, non-normal distribution). In this study, bootstrap analysis was conducted with 1,000 replicates, a relatively high number, in order to generate accurate estimates of the standard errors.

A typical microarray experiment measures the expression of several thousand genes simultaneously across different conditions. When investigating for potential DEGs between two conditions, each gene is treated independently, and the *t*-test is performed on each gene separately. The incidence of false positives (i.e., genes falsely declared as DEGs) is proportional to the number of tests performed and the critical significance level (*p*-value cut-off). In order to account for multiple comparisons, a correction method proposed by Benjamini and Hochberg (1995) which controls False Discovery Rate (FDR) was applied. FDR-controlling procedures have greater power (i.e., they can discover more statistically significant differences), at the expense of increased Type I error rate. Genes with adjusted *p*-value (or *q*-value) less or equal to 0.05 were considered as statistically significant in this study. For all statistical analyses, the Stata 13 statistical software package (StataCorp, 2013) was used. For the creation of heatmaps from microarray data, the average linkage clustering with Euclidean distance clustering method implemented in Heatmapper (http://heatmapper.ca/) was utilized.

### RNA-Seq-Based Transcriptomic Data Analysis

For the RNA-Seq based transcriptome analysis, the following pipeline was utilized. The FASTQ files were extracted from the respective Sequence Read Archive (SRA) files containing raw RNA-Seq reads by using the SRA Tool Kit v.2.9.0 (Alnasir and Shanahan, 2015) with the “*fastq-dump –gzip –skip-technical –readids –dumpbase –clip –split-3”* command. The raw RNA-Seq reads in FASTQ files were aligned to the human reference genome GRCh38 (Ensembl version 97) by employing the splice junction aligner HISAT2 v.2.1.0 (Kim et al., 2015) with “*hisat2 -p -dta -x {input.index} -U {input.fq} -S {out.sam}”* parameters. The generated SAM file was converted to the respective binary BAM file by using SAM Tools v.1.9.0 (Li et al., 2009) with “*samtools sort -@ 10 -o {output.bam} {input.sam}”* commands. String Tie v.1.3.5 (Pertea et al., 2015) was utilized with “*stringtie -e -B -p -G {input.gtf} -A {output.tab} -o {output.gtf} -l {input.label}{input.bam}”* parameters for the measurement of gene expression levels. The reconstructed transcripts and transcript abundances were reported in the output GTF file. In order to detect differentially expressed genes between RR and RS samples, we utilized the EdgeR package v3.28.0 (Robinson et al., 2010) of the R statistical computation environment v.3.6.1 (https://www.r-project.org). We firstly applied the trimmed mean of M-values (TMM) normalization (Robinson and Oshlack, 2010) implemented in EdgeR to the count data and we employed generalized linear models with “cell lines,” “resistance,” and “time” as factors. Then, estimating dispersion was computed with the *estimateDisp* function, and differential expression analysis between the two RNA-Seq groups (RR and RS) was performed using the *glmFit* and *glmLRT* functions of the EdgeR package v3.28.0 (Robinson et al., 2010) of the R statistical computation environment v.3.6.1 (https://www.r-project.org). In order to detect statistically significant differentially expressed genes, the threshold for the absolute log_2_-fold change was set at two (|log_2_FC≥2|), and for the FDR (Benjamini and Hochberg, 1995)-corrected *p*-value at 0.05. All statistical calculations for the RNASeq data were performed by using the R software environment. The *pheatmap* package of R (https://CRAN.R-project.org/package=pheatmap) was utilized to generate a heatmap from RNA-Seq data.

### Pathway Enrichment Analysis

To assign biological role(s) to the genes under study that are associated with biological pathways, gene set enrichment analysis (GSEA), or functional enrichment analysis, was conducted. GSEA is a method to identify biological processes or pathways that are over-represented in a large set of genes. To this end, WebGestalt (WEB-based GEne SeT AnaLysis Toolkit) (Zhang et al., 2005; Liao et al., 2019) was employed to identify statistically significant over-represented WikiPathways (Kutmon et al., 2016) cancer terms in the sets of genes; the threshold for the FDR-corrected *p*-value was set at 10^−3^, and hypergeometric distribution analysis was used.

### Functional Interactions Networks

The associations among the molecules under study were investigated and visualized with the usage of STRING (Search Tool for Retrieval of Interacting Genes/Proteins) v11.0 (Szklarczyk et al., 2019), a database of either known or predicted, direct or indirect, gene/protein associations derived from diverse resources. The highest confidence interaction score (≥0.9) was chosen to display the associations amongst genes/proteins in the generated network.

### Survival Analysis

The prognostic potential of the 36 radiogenes was investigated in three types of cancers, namely, breast invasive carcinoma (BRCA), colon adenocarcinoma (COAD), and acute myeloid leukemia (LAML), a type of haematologic cancer like ALL, through the web-based tool GEPIA (Gene Expression Profiling Interactive Analysis) (Tang et al., 2017) version 2 (http://gepia2.cancer-pku.cn/#index), based on data acquired from The Cancer Genome Atlas (TCGA) (https://tcga-data.nci.nih.gov). The cancer patient cohort is divided into the high-risk and low-risk categories; the cut-offs for low and high gene expression level patient cohorts were set at 50%.

### Tissue-Wise Differential Gene Expression Analysis

GEPIA2 (Tang et al., 2017), which contains gene expression data from cancer and corresponding normal tissues from TCGA (https://tcga-data.nci.nih.gov) and the Genotype-Tissue Expression (GTEx) (https://gtexportal.org/home/), respectively, was used to investigate the differential expression patterns of the radiogenes under study in BRCA, COAD, and LAML cancer-normal tissue; |logFC| ≥ 2 and FDR-corrected *p-*value ≤ 0.05.

## Results

**Figure 1:**
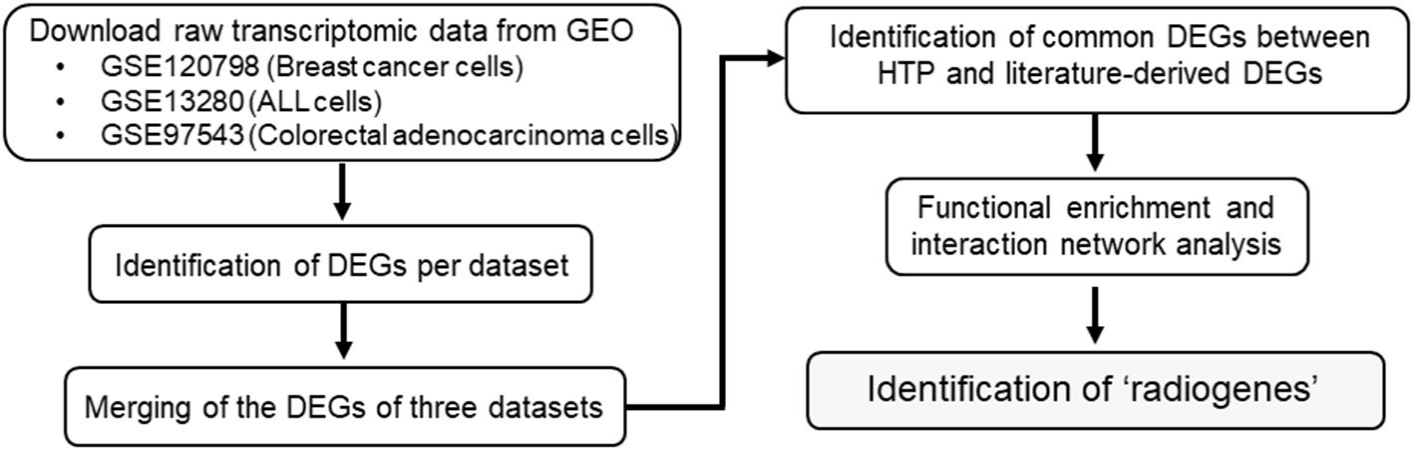
Flowchart diagram of the overall methodology employed in this study. Three eligible transcriptome datasets were retrieved from NCBI's GEO. Genes differentially expressed (DEGs) between the radioresistant and radiosensitive cancer cells were identified per dataset. The DEGs of the three analyzed datasets were integrated and compared with an earlier own study on cancer cell radioresistance-related genes/proteins derived from literature research. Functional enrichment analysis of the common genes was performed to obtain the so-called “radiogenes”.

The overall procedure for data collection and analysis followed in the present study is described illustratively in Figure 1.

### Identification of Differential Expression Patterns in Radioresistance vs. Radiosensitive Cancer Cells

The two techniques for transcriptome profiling, RNA-Seq and microarray, have large inherent differences. RNA-Seq is considered “superior” since it allows the detection of low abundance transcripts and novel transcript isoforms (Marioni et al., 2008). For this reason, we applied different statistical methods for RNA-Seq (Li, 2019) and microarray (Kontou et al., 2018) data processing and analysis.

**Figure 2:**
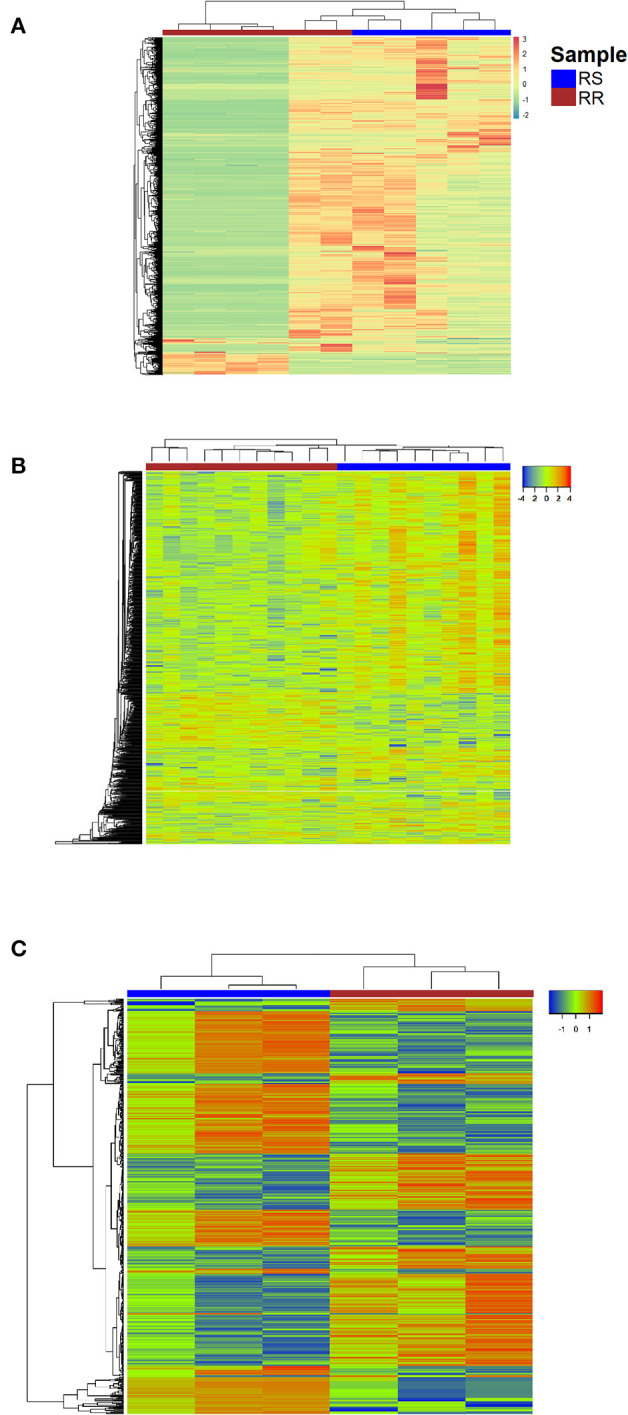
Heatmap representing color-coded expression levels of DEGs for the datasets **(A)** GSE120798 (breast cancer), **(B)** GSE13280 (ALL), **(C)** GSE97543 (COAD); columns correspond to samples and rows correspond to genes. RR, Radioresistant; RS, Radiosensitive.

**Figure 3:**
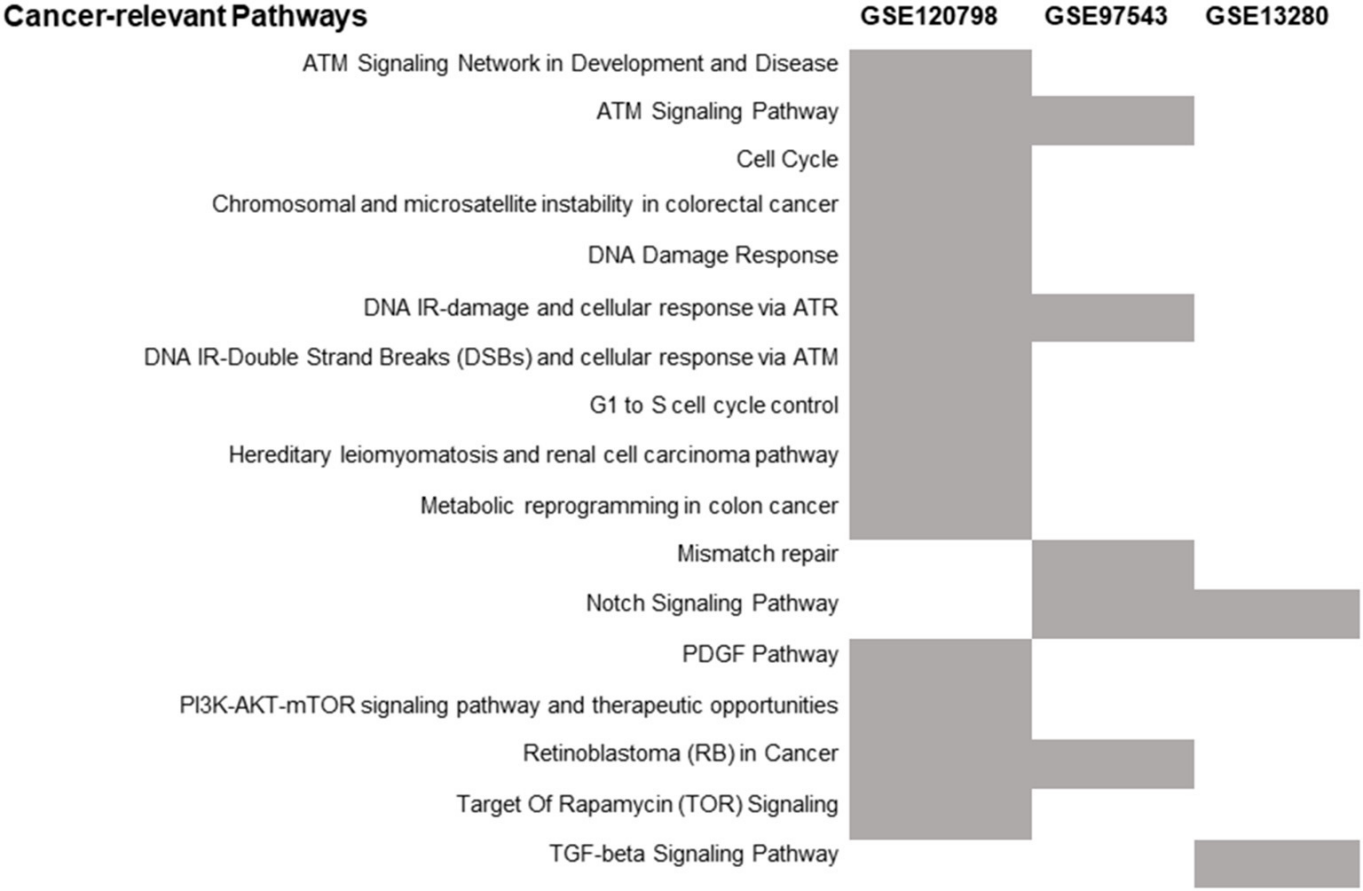
Distribution of the over-represented cancer-related WikiPathways in the DEGs of each transcriptome dataset. The enriched pathways are indicated by gray.

The number of differentially expressed genes (DEGs) found between radioresistant and radiosensitive cancer cells (Figure 2) for each dataset is 6372 (GSE120798), 782 (GSE13280), and 541 (GSE97543), respectively (Supplementary Table 2). The pathways over-represented in the DEGs of the three datasets are related primarily to DDR/R and cell survival (Figure 3).

Differential gene expression analysis was performed for the three parental breast cancer cell lines in GSE120798 (Gray et al., 2019) against their radioresistant derivatives, to identify those genes that are significantly dysregulated in response to radiation stress. A total of 11 samples were compared; one biological replicate for each condition (Supplementary Table 1). The number of detected DEGs (Gray et al., 2019) is remarkably higher as compared to the ones derived from microarray gene expression data (Supplementary Table 2). This discrepancy is likely due to the ability of RNA-Seq to detect and quantify, even rare, transcripts without *a priori* knowledge of a given gene (Metzker, 2010). Accordingly, the number of enriched pathways is also greater in this dataset (Figure 3). Three DDR/R pathways were found to be enriched in the DEGs, including the generic “DNA Damage Response” pathway. Two pathways are specifically implicated in IR-induced DNA damage response, namely “DNA IR-Double Strand Breaks (DSBs) and cellular response via ATM,” and “DNA IR-damage and cellular response via ATR.” Ataxia telangiectasia mutated (ATM) and ataxia telangiectasia and Rad3-related (ATR) proteins are evolutionarily conserved proteins that have a critical regulatory role in DDR and maintenance of genome integrity (Marechal and Zou, 2013; Awasthi et al., 2015). Furthermore, the “PI3K-AKT-mTOR signaling pathway and therapeutic opportunities” was shown to be over-represented in the set of DEGs; increased activity of PI3K in the radioresistant cells of this transcriptome dataset was also observed by Gray et al. (2019). Phosphatidylinositol-3-kinase (PI3K)/AKT/mammalian target of rapamycin (PI3K/AKT/mTOR) signaling is critical to many aspects of tumor cell growth and survival (Porta et al., 2014) and therefore could be likely involved in the survival of irradiated cancer cells.

Differential expression analysis was also carried out of the irradiated COAD cells in the GSE97543 dataset (Emons et al., 2017). A total of six samples were compared; three biological replicates for the wild-type (radiosensitive) cell lines and three for the radioresistant cells (Supplementary Table 1). The notch signaling pathway is significantly over-represented (Figure 3) in the DEGs of this dataset (Supplementary Table 2). Notch signaling is suggested to confer a selective survival advantage on tumors (Capaccione and Pine, 2013). Hence, the Notch network could be implicated in the resistance and survival of the COAD cells to irradiation. Regarding the over-represented DDR/R pathways, one pathway is related to processing IR-induced DNA lesions through ATR signaling and the other to mismatch repair (MMR), which is responsible for detecting and repairing mismatched nucleotides (Iyer et al., 2006; Larrea et al., 2010). MMR reaction is initiated by binding of the MSH2 (MutS homolog 2)/MSH6 heterodimer to the mismatched DNA; both *MSH2* and *MSH6* were found differentially expressed in the radioresistant COAD cells. The MutS homologs MSH2 and MSH6 form a heterodimer that binds to short insertion/deletion DNA mispairs (Habraken et al., 1996; Edelbrock et al., 2013). The MMR proteins also function in signaling DNA damage (Duckett et al., 1996b; Modrich, 1997). Earlier studies have shown that MSH2 is also involved in the processing of the biologically significant clustered DNA damages, as well as the execution of apoptosis induced by IR (Holt et al., 2009).

In GSE13280 (Marston et al., 2009), the gene expression profiles of radioresistant and radiosensitive ALL cell lines (eleven replicates for each condition) were compared (Supplementary Table 1) for the identification of DEGs (Supplementary Table 2). The enriched transforming growth factor beta (TGFB) signaling pathway (Figure 3), like the Notch network (Capaccione and Pine, 2013), is implicated in several aspects of cancer initiation, promotion, and progression (Syed, 2016). Hence, the Notch- and TGFB-mediated signaling pathways might render ALL cells less vulnerable to IR-induced apoptosis by exerting their cellular pro-survival effect.

### Identification of Cancer Cell Radioresistance–Related Genes

**Figure 4:**
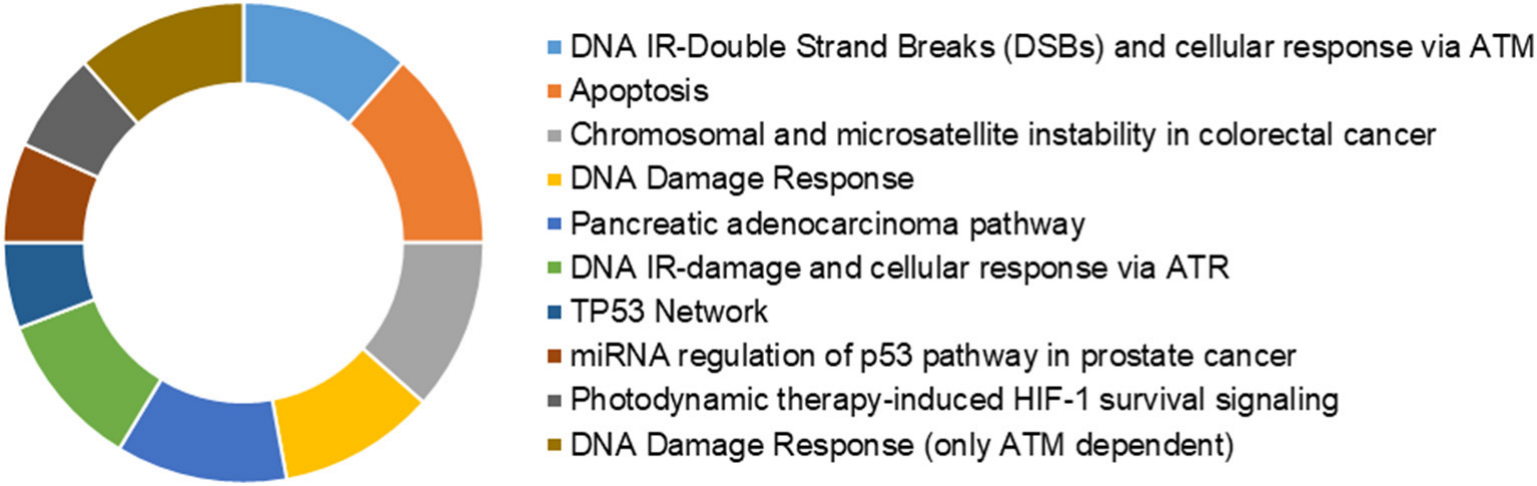
Donut chart depicting the over-represented cancer-relevant pathways across the 88 common genes.

**Figure 5:**
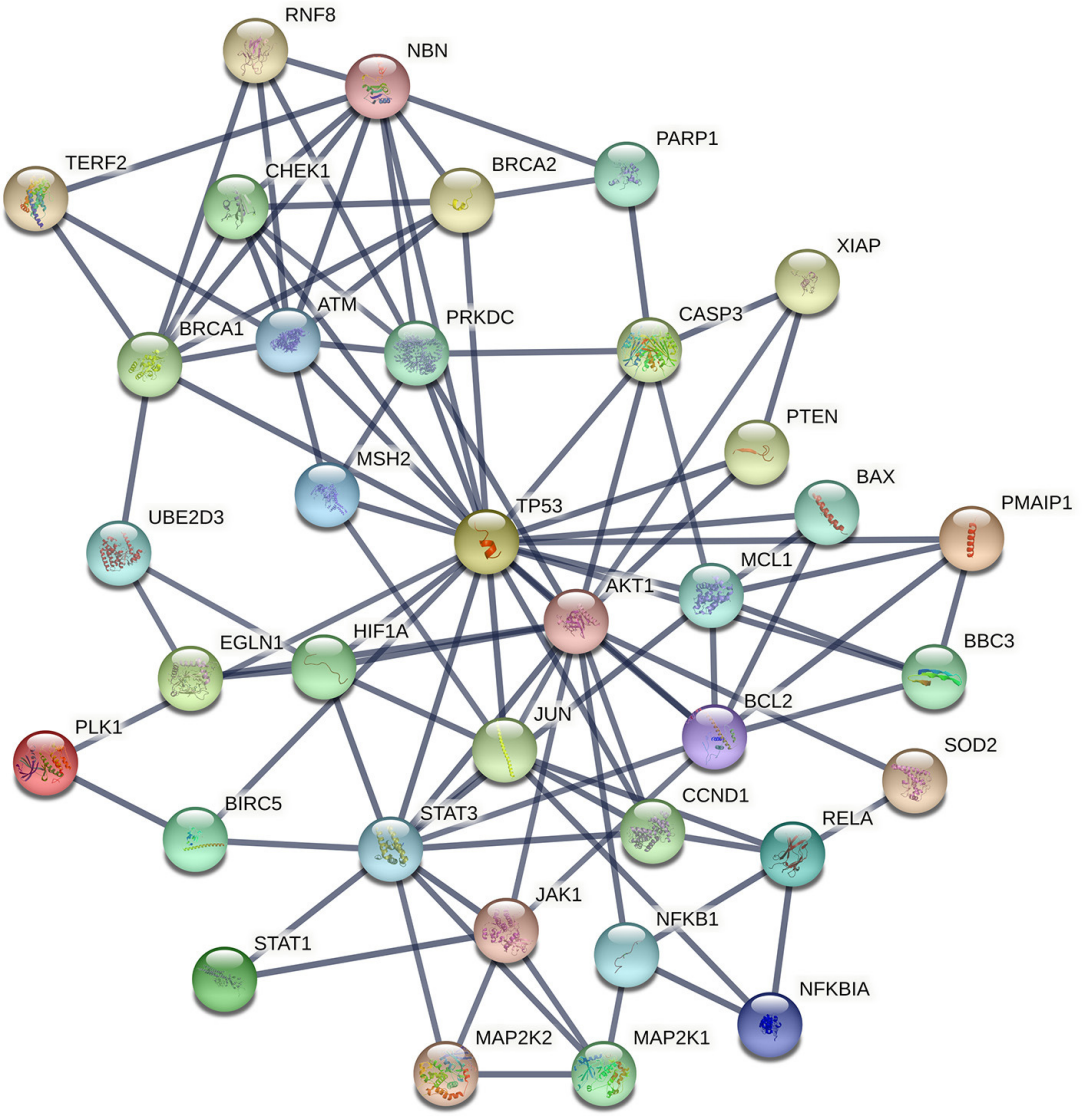
STRING interaction network of the products of the 36 radiogenes. The nodes represent proteins and the edges indicate different modes of interactions with a confidence score ≥ 0.9.

The procedure we followed to identify an optimal number of cancer cell radioresistance–related genes is illustrated in Supplementary Figure 1. A list of 175 bio molecules (Supplementary Table 3) was proposed in a previous study by Pavlopoulou et al. (2017) to be implicated in tumor cell radioresistance. These genes/proteins were manually collected through a comprehensive and thorough literature search. The DEGs identified in each of the three transcriptome datasets were merged; a list of 7,185 genes was compiled (Supplementary Table 3). Those genes were compared to the literature-derived molecules, and 88 genes were found in common (Supplementary Table 3). In order to identify an optimal number of genes implicated in radioresistance, we performed functional enrichment analysis of these genes. The pathways over-represented in the 88 genes (Figure 4) are related to DDR/R, similar to those detected in the DEGs of the individual datasets (Figure 3), as well as to apoptosis. Signaling pathways mediated by the cardinal tumor suppressor TP53 are also related to radioresistance (Figure 4). Collectively, 36 genes were found to be implicated in cancer-associated biological pathways listed in Table 1, 26 of those up-regulated and 10 down-regulated in RR cancer cells. The products of the 36 genes also form a highly connected network (Figure 5), with high-confidence interactions, suggesting that these proteins associate, either functionally or physically, to confer cellular resistance to IR. Therefore, we propose 36 interconnected pivotal genes, henceforth referred to as “radiogenes,” which participate in radioresistance-relevant pathways and mechanisms.

**Table 1:** Gene symbol and expression status of the 36 differentially expressed radiogenes (radioresistant vs. radiosensitive cancer cells).

**Gene**	**Expression status**	**Cell line**
*AKT1*	Up	BC
ATM	Up	BC
BAX	Down	BC; ALL
BBC3	Down	BC
*BCL2*	Up	BC
*BIRC5*	Up	BC
*BRCA1*	Up	BC
*BRCA2*	Up	BC
CASP3	Down	BC
*CCND1*	Up	BC
*CHEK1*	UP	BC
EGLN1	Up	BC
*HIF1A*	Up	BC; ALL
*JAK1*	Down	BC
JUN	Up	BC
MAP2K1	UP	BC
MAP2K2	Up	BC
MCL1	Up	BC
*MSH2*	Up	BC; COAD
*NBN*	Up	BC
*NFKB1*	Up	BC
*NFKBIA*	Down	BC
*PARP1*	Up	BC
*PLK1*	Up	BC
PMAIP1	Down	BC
*PRKDC*	Up	BC
*PTEN*	Down	BC
RELA	Up	BC
*RNF8*	Up	BC
SOD2	Up	BC; ALL
STAT1	Down	BC
STAT3	Up	BC
TERF2	Up	BC
TP53	Down	BC
UBE2D3	Down	ALL
*XIAP*	Up	BC

*ALL, acute lymphoblastic leukemia; BC, breast cancer; COAD, colorectal adenocarcinoma*.

*Those radiogenes found to be differentially expressed between TCGA-derived cancer tissue and corresponding GTEx matched normal tissue are italicized*.

### Differentially Expressed Radiogenes in Cancer vs. Normal Tissue

Collectively, 19 radiogenes, found to be differentially expressed in the three radioresistant vs. the radiosensitive cancer cell lines (Table 1), are consistently deregulated in their corresponding cancer-matched normal tissues with the same direction (i.e., up- or down-regulated) (Supplementary Figure 2). This finding could be utilized in personalized tumor treatment for the selective eradication of cancer cells by applying radiotherapy without harming the adjacent healthy tissue at the same time.

### Radiogenes Are Potential Cancer Prognostic Markers

The differential expression of the radiogenes can also predict the clinical outcome of cancer patients. In particular, a statistically significant association was found between *CHEK1, MAP2K1*, and *PLK1* overexpression and worse overall survival in breast invasive carcinoma patients, indicated by pooled hazard ratio (HR) values higher than 1 and *p*-values lower than 0.05. Conversely, a significant relationship was observed between high expression of *NFKBIA*, which is otherwise underexpressed in radioresistant breast cancer cells, and favorable prognosis, indicated by an HR value <1 (Supplementary Figure 3).

## Discussion

Cancer cells confer resistance to irradiation through diverse mechanisms including enhanced DNA damage repair capacity, activation of cell survival signaling pathways, inhibition of apoptotic pathways, and induced autophagy.

DDR/R is a complex entangled process consisting of recognition (or detection, or sensing), signaling, and repair of DNA damage (Rouse and Jackson, 2002). Ionizing radiation usually generates a variety of DNA lesions, including abasic (apurinic/apyrimidinic) sites, oxidized bases, crosslinks between thymine and cytosine bases, DNA single-strand breaks (SSBs), and DNA double-strand breaks (DSBs) (Sutherland et al., 2000). In the case of SSBs, only one strand of the double stranded DNA helix is severed. SSBs are recognized and processed by base excision repair (BER) and nucleotide excision repair (NER) mechanisms (Caldecott, 2008). DSB is the most detrimental type of DNA lesions since both strands of the double helix are broken. DSBs are repaired mainly through homologous recombination (HR) if cells are present in the S/G2 cell-cycle phase or, the less accurate, non-homologous end joining (NHEJ) (Budman and Chu, 2005). Those different types of DNA lesions can be formed either separately or in close vicinity (a few nm), resulting in clustered DNA lesions or locally multiply damaged sites (MDS). Clustered DNA damage, the hallmark of IR, is considered the most severe type of genomic damage because of its complexity. This complex DNA damage includes both DSBs and non-DSBs, usually referred to as oxidatively-clustered DNA lesions (OCDLs), occurring within a DNA region of 15–20 bp. The corresponding DNA damage repair mechanisms are recruited by the cell in response to clustered damaged sites. However, harnessing the corresponding DNA repair machinery to the clustered damaged sites is quite challenging since the presence of a lesion in one strand can delay significantly the simultaneous processing of another lesion on the complementary strand. Furthermore, OCDLs can be rapidly converted into *de novo* DSBs by a DNA glycosylase during the repair. As a result, unrepaired MDS can lead to increasing levels of genotoxic damage, triggering also systemic responses (Nikitaki et al., 2016). Clustered DNA lesions, if not properly processed, could contribute to increased genomic instability in the form of chromosomal aberrations (e.g., deletions and inversions) and microsatellite instability, leading eventually to carcinogenesis. Therefore, the induction of clustered DNA damage increases the cytotoxic effect of radiation, especially in highly proliferating cancer cells. Radioresistant cancer cells though can counteract this effect through their ability to respond to and repair complex/clustered DNA damage more efficiently without avoiding necessarily increased genomic instability, as compared to their radiosensitive counterparts (Hada and Georgakilas, 2008; Georgakilas et al., 2013; Mavragani et al., 2017, 2019; Bukowska and Karwowski, 2018). The radiogenes identified in the present study are implicated in diverse mechanisms underlying the acquisition of radioresistance in cancer cells.

Among the 36 “radiogenes”, *ATM, BRCA1/2, CHEK1, CCND1, MSH2, NBN, PARP1, PLK1, PRKDC*, and *RNF8*, which are consistently up-regulated across the radioresistant cancer cell lines (Table 1) and the corresponding cancer tissue (Supplementary Figure 2), are crucially implicated in different stages of DDR/R. In particular, ATM plays a protagonistic role in the initial stage of DDR/R, that is, DNA damage detection and stress-response signaling (Iliakis et al., 2003; Yang et al., 2004). ATM signaling is activated by a wide variety of DNA lesions, as well as DNA replication stress (Marechal and Zou, 2013; Burger et al., 2019). Mutations in ATM result in the genetic disorder ataxia-telangiectasia (AT), which is characterized by the high sensitivity of AT patients to IR and cancer predisposition (Gatti et al., 1988). Checkpoint kinase 1 (CHEK1), which acts downstream of ATM, is a core regulator of cell cycle checkpoint signaling in DNA damage response (Flaggs et al., 1997; Patil et al., 2013). Cyclin D1 (CCND1) was demonstrated to induce post-DNA damage cell cycle arrest and apoptosis in different types of cancers (Cai et al., 2012; Smith et al., 2016). PRKDC is a serine/threonine DNA-activated protein kinase involved in DSB recognition and DNA damage repair through NHEJ (Soubeyrand et al., 2003). Notably, ATM and PRKDC were found to affect greatly cancer cell response to IR through genome-wide genetic screening in a recent study by Francica et al. (2020). Moreover, BRCA1 and BRCA2 are largely involved in cell cycle control and maintenance of genomic stability in response to DNA damage (Deng, 2006; Gudmundsdottir and Ashworth, 2006). Another radiogene, *NBN (nibrin)*, encodes one of the three components of the MRN complex (MRE11A-RAD50-NBN), which is implicated in the recognition and repair of DSBs (Lamarche et al., 2010). NBN is mutated in patients with Nijmegen breakage syndrome (NBS), and cells from NBS patients are hypersensitive to IR (Taalman et al., 1983). In addition, a functional link between ATM and NBN proteins has been demonstrated by Zhao et al. (2000). Also, the MMR protein MSH2 (MutS homolog 2) is suggested to contribute to radioresistance via SSB processing (Li et al., 2016). Moreover, RNF8 (ring finger protein 8) protein catalyzes the mono-ubiquitination of histones H2A and H2B during DNA damage, thereby facilitating DNA damage repair and activation of cell cycle checkpoint (Kolas et al., 2007; Ma et al., 2011); RNF8 is associated with radioresistance in human nasopharyngeal cancer cells (Wang et al., 2015). The protein encoded by the radiogene *PLK1 (Polo-like kinase 1)* is involved in cell cycle resumption following DNA damage-induced checkpoint arrest (Hyun et al., 2014; Bruinsma et al., 2017). It has been demonstrated that inhibition of *PLK1* renders glioblastoma and non-small cell lung cancer cells sensitive to IR (Pezuk et al., 2013; Van den Bossche et al., 2019). Of particular note, pharmacological inhibitors of the BER enzyme PARP1 [poly(ADP-ribose) polymerase 1], such as niraparib, olaparib, and rucaparib, are widely used for targeted cancer therapy (Sulai and Tan, 2018; Patel et al., 2020). Importantly, *CHEK1* and *PLK1* were found to be poor prognostic biomarkers for the survival of breast cancer patients (Supplementary Figure 3), further supporting that enhanced DNA damage repair mechanisms in cancer cells play a catastaltic role in efficient radiotherapy (Pavlopoulou et al., 2016, 2017). Therefore, DDR/R might represent a primary “danger” signal, leading eventually to the activation of downstream signaling cascades and pro-survival mechanisms (Nikitaki et al., 2015).

The apoptotic pathway is also affected by the cellular response to radiation-induced genomic damage in cancers, as we suggest in this study. Radioresistant cancer cells have developed the ability to evade apoptosis prompted by their response to extreme and repair-resistant DNA damage, mainly due to deregulation of key pro-apoptotic molecules like TP53 (Fridman and Lowe, 2003; Haupt et al., 2003), PTEN (Lu et al., 2016), PMAIP1 (mediator of damage-induced p53-mediated apoptosis) (Oda et al., 2000), BBC3 (TP53-upregulated modulator of apoptosis) (Han et al., 2001; Nakano and Vousden, 2001), BAX (BCL2-associated X protein) (Pawlowski and Kraft, 2000), as well as anti-apoptotic proteins like MCL1 (Fujise et al., 2000; Glaser et al., 2012), BCL2 (Akl et al., 2014), BIRC5 (Chiou et al., 2003), and XIAP (X-linked inhibitor of apoptosis) (Duckett et al., 1996a; Deveraux and Reed, 1999). In a consistent manner, in this study, the pro-apoptotic genes were shown to be up-regulated, whereas the anti-apoptotic genes are down-regulated in radioresistant cancer cells (Table 1). Of those, BCL2, which is down-regulated in radioresistant breast cancer cells and tissue (Table 1, Supplementary Figure 2), can suppress apoptosis by inhibiting the activity of caspases indispensable for apoptosis, such as caspase-3 (CASP3) (Porter and Janicke, 1999; Swanton et al., 1999). Of note, aberrant activation of the Notch signaling pathway was demonstrated to inhibit TP53-mediated damage response and promote breast carcinogenesis by preventing apoptosis (Stylianou et al., 2006), suggesting a link between pro-survival and apoptotic mechanisms. PTEN was also found to promote apoptosis and cell cycle arrest via PI3K/AKT-dependent and -independent signaling (Weng et al., 2001).

The radiogene *NFKB1*, which transactivates several pro-inflammatory genes (Liu et al., 2017), was found to be overexpressed in radioresistant breast cancer cells and tissue (Table 1, Supplementary Figure 2). Furthermore, the members of the NFKB1 family, NFKBIA and RELA, can act either as inducers or inhibitors of apoptosis, respectively (Sonenshein, 1997; Barkett and Gilmore, 1999), consistent with their expression status in RR cells (Table 1; over- and under-expressed). The well-known NFKB1 inhibitor alpha (NFKBIA) was shown to be a favorable predictor in breast cancer patients (Supplementary Figure 3). Moreover, NFKB1, together with the inflammatory factors HIF1A (hypoxia-inducible factor 1) and STAT3, both of which were found to be up-regulated in radioresistant breast cancer cells (Table 1) and HIF1A overexpressed in breast cancer tissue (Supplementary Figure 2), are critically implicated in cancer radioresistance and radiation-induced inflammatory responses (Multhoff and Radons, 2012). On the other hand, STAT1, found down-regulated in the same cell lines (Table 1), as opposed to STAT3, elicits pro-apoptotic and anti-proliferative responses and promotes anti-cancer immunity (Avalle et al., 2012).

Suppression of *UBE2D3*, which is down-regulated in radioresistant ALL cells (Table 1), was demonstrated in a study by Wang et al. (2013) to decrease radiosensitivity of human breast cancer cells by promoting telomere maintenance. In addition, UBE2D3 is negatively correlated with TERF2 (telomeric repeat binding factor 2), the latter of which is primarily involved in telomere maintenance (Kim et al., 2009) and is down-regulated in radioresistance (Table 1).

Radiation can also exert its genotoxic and cytotoxic effects through the indirect and systemic induction of severe oxidative stress and the production of ROS in the organism (Kryston et al., 2011). The radiogene *HIF1A* plays a pivotal cytoprotective role against oxidative stress (Li et al., 2019) by inhibiting autophagy and cell death (Pouyssegur et al., 2006). Moreover, the superoxide scavenging enzyme SOD2 (superoxide dismutase 2), found overexpressed both in radioresistant breast cancer and ALL cells (Table 1), at normal expression levels provides a cytoprotective effect. Thus, SOD2 can likely exert a protective effect on RR cancer cells by controlling potential ROS-mediated DNA damage via catalyzing the reduction of superoxide into less genotoxic molecules like oxygen (Wang et al., 2018).

Notably, in this study, pro-survival pathways (Figure 3) like Notch signaling, were found to be implicated both in solid and blood cancers, probably by mediating survival of cancer cells to radiation-induced clustered/complex DNA damage (Marston et al., 2009; Capaccione and Pine, 2013). Moreover, the PI3K/AKT/mTOR signaling pathway is suggested to be important in regulating cell survival in response to different types of cellular stress (Hung et al., 2012; Porta et al., 2014), including genotoxic stress. Hence, pro-survival pathways could be considered as potential therapeutic targets in cancer (Porta et al., 2014).

A major limitation of this study, particularly for the two solid cancers datasets, is the lack of patient-derived tumor tissue, as well as cancer stem cells, which constitute a subpopulation in solid tumors that display stem-like characteristics (Pavlopoulou et al., 2016). Instead, the respective experimental studies relied on the use of commercial cancer cell lines; of particular note, the ALL cells were derived from “real” patients. However, the extent to which the individual cancer cell lines can capture the cellular and genomic complexity of tumors is questioned. Further research is needed to determine whether the results derived from the cancer cell lines investigated in this study or other studies could be extrapolated to their corresponding tissues of origin, like breast tumor tissue and colorectal adenocarcinoma. Nevertheless, it is suggested that large panels of cancer cell lines can faithfully capture the genomic heterogeneity of cancers (Sakellaropoulos et al., 2019; Vougas et al., 2019). Beyond the discussed limitations, the over- or under-expressions of the radiogenes in radioresistant phenotypes have been verified, to a great extent, by independent experimental biochemical studies available in the literature. In addition, the clinical implications of those genes are further supported by the patient survival results (Supplementary Figure 3).

## Conclusion

In the present study, we employed an integrative bioinformatics approach to analyze transcriptomic data regarding the molecular determinants of cancer cell radioresistance. On the basis of our findings, both solid and hematologic cancer cells likely depend on similar mechanisms to confer resistance to IR (i.e., DDR/R and cell survival). Moreover, we identified 36 functionally associated radiogenes that participate in radioresistance-associated pathways. Most of those radiogenes were also shown to be differentially regulated in the corresponding cancer tissues. Moreover, several of the radiogenes were found to have potential prognostic value for the clinical outcome of cancer patients. However, the availability of clinically derived cancer tissues would provide a more reliable source for conducting research on the response of cancer patients to radiation. The overall data presented herein can be particularly useful for clinicians in selecting suitable targets (e.g., DDR/R inhibitors) for appropriate combination therapy using IR. In conclusion, we suggest that this bioinformatics premise can be harnessed as a first step in the rational design of *in vivo* experimental studies or in personalized medicine for optimizing tumor response and cancer cell susceptibility to therapeutic ionizing radiation and reduction of the total effective radiation dose administered to the patient.

## Data Availability Statement

The original contributions presented in the study are included in the article/Supplementary Material; further inquiries can be directed to the corresponding author/s.

## Author Contributions

AP conceived the study. AP and AGG designed and supervised the study. HIT, GK, PK, and AP performed the experiments and analyzed the data. HIT, GK, PK, HA, AGG, and AP wrote the manuscript. All authors reviewed and approved of the final manuscript.

## Conflict of Interest

The authors declare that the research was conducted in the absence of any commercial or financial relationships that could be construed as a potential conflict of interest.

## Supplementary Material

Supplementary Figure 1 Schematic workflow outlining the selection of radiogenes. Venn diagram comparing the high-throughput (HTP) and literature-derived cancer radioresistance-associated genes. Functional enrichment and interaction network analysis of the common 88 genes resulted in 36 genes. Red, up-regulated genes; Green, down-regulated genes.

Supplementary Figure 2 Differential expression patterns of radiogenes in the corresponding cancer tissue; BRCA, breast invasive carcinoma; COAD, colon adenocarcinoma; LAML, acute myeloid leukemia. The red box represents cancer tissue samples and the gray box indicates normal samples.

Supplementary Figure 3 Kaplan-Meier curves depicting the prognostic value of radiogenes for overall survival in invasive breast carcinoma **(A)** *CHEK1*, **(B)** *PLK1*, **(C)** *MAP2K1* and **(D)** *NFKBIA*. The HR “HR(high)” and the corresponding p-values “p(HR)” are shown. The 95% confidence intervals (CI) are denoted by dotted lines. The number of high-risk and low-risk patients are indicated by “n(high)” and “n(low),” respectively.

Supplementary Table 1 Samples from each transcriptomic dataset analyzed in this study.

Supplementary Table 2 List of the differentially expressed genes (DEGs) of each of the three transcriptome datasets and the DEGs common among datasets. Log_2_FC of radioresistant vs. radiosensitive differentially expressed genes in GSE120798 (breast cancer) cell lines; cut-off |log_2_FC≥2|.

Supplementary Table 3 List of the merged DEGs from each dataset, literature-derived genes/proteins and common molecules.

## References

[B1] Akl H. Vervloessem T. Kiviluoto S. Bittremieux M. Parys J. B. De Smedt H.. (2014). A dual role for the anti-apoptotic Bcl-2 protein in cancer: mitochondria versus endoplasmic reticulum. Biochim. Biophys. Acta 1843, 2240–2252. 10.1016/j.bbamcr.2014.04.017 24768714

[B2] Alnasir J. Shanahan H. P. (2015). Investigation into the annotation of protocol sequencing steps in the sequence read archive. Gigascience 4:23. 10.1186/s13742-015-0064-7 25960871 PMC4425880

[B3] Avalle L. Pensa S. Regis G. Novelli F. Poli V. (2012). STAT1 and STAT3 in tumorigenesis: a matter of balance. JAKSTAT 1, 65–72. 10.4161/jkst.20045 24058752 PMC3670295

[B4] Awasthi P. Foiani M. Kumar A. (2015). ATM and ATR signaling at a glance. J. Cell Sci. 128, 4255–4262. 10.1242/jcs.169730 26567218

[B5] Barkett M. Gilmore T. D. (1999). Control of apoptosis by Rel/NF-kappaB transcription factors. Oncogene 18, 6910–6924. 10.1038/sj.onc.1203238 10602466

[B6] Barrett T. Wilhite S. E. Ledoux P. Evangelista C. Kim I. F. Tomashevsky M.. (2013). NCBI GEO: archive for functional genomics data sets–update. Nucleic Acids Res. 41, D991–D995. 10.1093/nar/gks1193 23193258 PMC3531084

[B7] Baskar R. Lee K. A. Yeo R. Yeoh K. W. (2012). Cancer and radiation therapy: current advances and future directions. Int. J. Med. Sci. 9, 193–199. 10.7150/ijms.3635 22408567 PMC3298009

[B8] Begg A. C. Stewart F. A. Vens C. (2011). Strategies to improve radiotherapy with targeted drugs. Nat. Rev. Cancer 11, 239–253. 10.1038/nrc3007 21430696

[B9] Beheshti A. McDonald J. T. Miller J. Grabham P. Costes S. V. (2019). GeneLab database analyses suggest long-term impact of space radiation on the cardiovascular system by the activation of FYN through reactive oxygen species. Int. J. Mol. Sci. 20:661. 10.3390/ijms20030661 30717456 PMC6387434

[B10] Benjamini Y. Hochberg Y. (1995). Controlling the false discovery rate: a practical and powerful approach to multiple testing. J. R. Stat. Soc. Ser. B 57, 289–300. 10.1111/j.2517-6161.1995.tb02031.x

[B11] Braschi B. Denny P. Gray K. Jones T. Seal R. Tweedie S.. (2019). Genenames.org: the HGNC and VGNC resources in 2019. Nucleic Acids Res. 47, D786–D792. 10.1093/nar/gky930 30304474 PMC6324057

[B12] Bruinsma W. Aprelia M. Garcia-Santisteban I. Kool J. Xu Y. J. Medema R. H. (2017). Inhibition of Polo-like kinase 1 during the DNA damage response is mediated through loss of Aurora A recruitment by Bora. Oncogene 36, 1840–1848. 10.1038/onc.2016.347 27721411 PMC5378932

[B13] Budman J. Chu G. (2005). Processing of DNA for nonhomologous end-joining by cell-free extract. EMBO J. 24, 849–860. 10.1038/sj.emboj.7600563 15692565 PMC549622

[B14] Bukowska B. Karwowski B. T. (2018). The clustered DNA lesions - types, pathways of repair and relevance to human health. Curr. Med. Chem. 25, 2722–2735. 10.2174/0929867325666180226110502 29484975

[B15] Burger K. Ketley R. F. Gullerova M. (2019). Beyond the trinity of ATM, ATR, and DNA-PK: multiple kinases shape the DNA damage response in concert with RNA metabolism. Front. Mol. Biosci. 6:61. 10.3389/fmolb.2019.00061 31428617 PMC6688092

[B16] Cai C. K. Zhao G. Y. Tian L. Y. Liu L. Yan K. Ma Y. L.. (2012). miR-15a and miR-16-1 downregulate CCND1 and induce apoptosis and cell cycle arrest in osteosarcoma. Oncol. Rep. 28, 1764–1770. 10.3892/or.2012.1995 22922827

[B17] Caldecott K. W. (2008). Single-strand break repair and genetic disease. Nat. Rev. Genet. 9, 619–631. 10.1038/nrg2380 18626472

[B18] Capaccione K. M. Pine S. R. (2013). The Notch signaling pathway as a mediator of tumor survival. Carcinogenesis 34, 1420–1430. 10.1093/carcin/bgt127 23585460 PMC3697894

[B19] Chiou S. K. Jones M. K. Tarnawski A. S. (2003). Survivin - an anti-apoptosis protein: its biological roles and implications for cancer and beyond. Med. Sci. Monit. 9, PI25–PI29. 12709681

[B20] Clough E. Barrett T. (2016). The gene expression omnibus database. Methods Mol. Biol. 1418, 93–110. 10.1007/978-1-4939-3578-9_5 27008011 PMC4944384

[B21] Delaney G. Jacob S. Featherstone C. Barton M. (2005). The role of radiotherapy in cancer treatment: estimating optimal utilization from a review of evidence-based clinical guidelines. Cancer 104, 1129–1137. 10.1002/cncr.21324 16080176

[B22] Deng C. X. (2006). BRCA1: cell cycle checkpoint, genetic instability, DNA damage response and cancer evolution. Nucleic Acids Res. 34, 1416–1426. 10.1093/nar/gkl010 16522651 PMC1390683

[B23] Deveraux Q. L. Reed J. C. (1999). IAP family proteins–suppressors of apoptosis. Genes Dev. 13, 239–252. 10.1101/gad.13.3.239 9990849

[B24] Duckett C. S. Nava V. E. Gedrich R. W. Clem R. J. Van Dongen J. L. Gilfillan M. C.. (1996a). A conserved family of cellular genes related to the baculovirus iap gene and encoding apoptosis inhibitors. EMBO J. 15, 2685–2694 10.1002/j.1460-2075.1996.tb00629.x 8654366 PMC450204

[B25] Duckett D. R. Drummond J. T. Murchie A. I. Reardon J. T. Sancar A. Lilley D. M.. (1996b). Human MutSalpha recognizes damaged DNA base pairs containing O6-methylguanine, O4-methylthymine, or the cisplatin-d(GpG) adduct. Proc. Natl. Acad. Sci. U. S. A. 93, 6443–6447. 10.1073/pnas.93.13.6443 8692834 PMC39042

[B26] Edelbrock M. A. Kaliyaperumal S. Williams K. J. (2013). Structural, molecular and cellular functions of MSH2 and MSH6 during DNA mismatch repair, damage signaling and other noncanonical activities. Mutat. Res. 743–744, 53–66. 10.1016/j.mrfmmm.2012.12.008 23391514 PMC3659183

[B27] Efron B. Tibshirani R. (1993). An Introduction to the Bootstrap. Boca Raton, FL: Chapman & Hall/CRC.

[B28] Emons G. Spitzner M. Reineke S. Moller J. Auslander N. Kramer F.. (2017). Chemoradiotherapy resistance in colorectal cancer cells is mediated by Wnt/beta-catenin signaling. Mol. Cancer Res. 15, 1481–1490. 10.1158/1541-7786.MCR-17-0205 28811361 PMC5772978

[B29] Flaggs G. Plug A. W. Dunks K. M. Mundt K. E. Ford J. C. Quiggle M. R.. (1997). Atm-dependent interactions of a mammalian chk1 homolog with meiotic chromosomes. Curr. Biol. 7, 977–986. 10.1016/S0960-9822(06)00417-9 9382850

[B30] Francica P. Mutlu M. Blomen V. A. Oliveira C. Nowicka Z. Trenner A.. (2020). Functional radiogenetic profiling implicates ERCC6L2 in non-homologous end joining. Cell Rep. 32:108068. 10.1016/j.celrep.2020.108068 32846126

[B31] Fridman J. S. Lowe S. W. (2003). Control of apoptosis by p53. Oncogene 22, 9030–9040. 10.1038/sj.onc.1207116 14663481

[B32] Fujise K. Zhang D. Liu J. Yeh E. T. (2000). Regulation of apoptosis and cell cycle progression by MCL1. Differential role of proliferating cell nuclear antigen. J. Biol. Chem. 275, 39458–39465. 10.1074/jbc.M006626200 10978339

[B33] Gatti R. A. Berkel I. Boder E. Braedt G. Charmley P. Concannon P.. (1988). Localization of an ataxia-telangiectasia gene to chromosome 11q22-23. Nature 336, 577–580. 10.1038/336577a0 3200306

[B34] Georgakilas A. G. O'Neill P. Stewart R. D. (2013). Induction and repair of clustered DNA lesions: what do we know so far? Radiat. Res. 180, 100–109. 10.1667/RR3041.1 23682596

[B35] Glaser S. P. Lee E. F. Trounson E. Bouillet P. Wei A. Fairlie W. D.. (2012). Anti-apoptotic Mcl-1 is essential for the development and sustained growth of acute myeloid leukemia. Genes Dev. 26, 120–125. 10.1101/gad.182980.111 22279045 PMC3273836

[B36] Gray M. Turnbull A. K. Ward C. Meehan J. Martinez-Perez C. Bonello M.. (2019). Development and characterisation of acquired radioresistant breast cancer cell lines. Radiat. Oncol. 14:64. 10.1186/s13014-019-1268-2 30987655 PMC6466735

[B37] Gudmundsdottir K. Ashworth A. (2006). The roles of BRCA1 and BRCA2 and associated proteins in the maintenance of genomic stability. Oncogene 25, 5864–5874. 10.1038/sj.onc.1209874 16998501

[B38] Habraken Y. Sung P. Prakash L. Prakash S. (1996). Binding of insertion/deletion DNA mismatches by the heterodimer of yeast mismatch repair proteins MSH2 and MSH3. Curr. Biol. 6, 1185–1187. 10.1016/S0960-9822(02)70686-6 8805366

[B39] Hada M. Georgakilas A. G. (2008). Formation of clustered DNA damage after high-LET irradiation: a review. J. Radiat. Res. 49, 203–210. 10.1269/jrr.07123 18413977

[B40] Han J. Flemington C. Houghton A. B. Gu Z. Zambetti G. P. Lutz R. J.. (2001). Expression of bbc3, a pro-apoptotic BH3-only gene, is regulated by diverse cell death and survival signals. Proc. Natl. Acad. Sci. U. S. A. 98, 11318–11323. 10.1073/pnas.201208798 11572983 PMC58727

[B41] Haupt S. Berger M. Goldberg Z. Haupt Y. (2003). Apoptosis - the p53 network. J. Cell Sci. 116 (Pt 20), 4077–4085. 10.1242/jcs.00739 12972501

[B42] Holt S. M. Scemama J. L. Panayiotidis M. I. Georgakilas A. G. (2009). Compromised repair of clustered DNA damage in the human acute lymphoblastic leukemia MSH2-deficient NALM-6 cells. Mutat. Res. 674, 123–130. 10.1016/j.mrgentox.2008.09.014 18955159

[B43] Hung C. M. Garcia-Haro L. Sparks C. A. Guertin A. D. (2012). mTOR-dependent cell survival mechanisms. Cold Spring Harb. Perspect. Biol. 4:a008771. 10.1101/cshperspect.a008771 23124837 PMC3504431

[B44] Hyun S. Y. Hwang H. I. Jang Y. J. (2014). Polo-like kinase-1 in DNA damage response. BMB Rep. 47, 249–255. 10.5483/BMBRep.2014.47.5.061 24667170 PMC4163859

[B45] Iliakis G. Wang Y. Guan J. Wang H. (2003). DNA damage checkpoint control in cells exposed to ionizing radiation. Oncogene 22, 5834–5847. 10.1038/sj.onc.1206682 12947390

[B46] Iyer R. R. Pluciennik A. Burdett V. Modrich P. L. (2006). DNA mismatch repair: functions and mechanisms. Chem. Rev. 106, 302–323. 10.1021/cr0404794 16464007

[B47] Kanakoglou D. S. Michalettou T. D. Vasileiou C. Gioukakis E. Maneta D. Kyriakidis K. V.. (2020). Effects of high-dose ionizing radiation in human gene expression: a meta-analysis. Int. J. Mol. Sci. 21:1938. 10.3390/ijms21061938 32178397 PMC7139561

[B48] Kim D. Langmead B. Salzberg S. L. (2015). HISAT: a fast spliced aligner with low memory requirements. Nat. Methods 12, 357–360. 10.1038/nmeth.3317 25751142 PMC4655817

[B49] Kim H. Lee O. H. Xin H. Chen L. Y. Qin J. Chae H. K.. (2009). TRF2 functions as a protein hub and regulates telomere maintenance by recognizing specific peptide motifs. Nat. Struct. Mol. Biol. 16, 372–379. 10.1038/nsmb.1575 19287395

[B50] Kolas N. K. Chapman J. R. Nakada S. Ylanko J. Chahwan R. Sweeney F. D.. (2007). Orchestration of the DNA-damage response by the RNF8 ubiquitin ligase. Science 318, 1637–1640. 10.1126/science.1150034 18006705 PMC2430610

[B51] Kontou P. I. Pavlopoulou A. Bagos P. G. (2018). Methods of analysis and meta-analysis for identifying differentially expressed genes. Methods Mol. Biol. 1793, 183–210. 10.1007/978-1-4939-7868-7_12 29876898

[B52] Kryston T. B. Georgiev A. B. Pissis P. Georgakilas A. G. (2011). Role of oxidative stress and DNA damage in human carcinogenesis. Mutat. Res. 711, 193–201. 10.1016/j.mrfmmm.2010.12.016 21216256

[B53] Kutmon M. Riutta A. Nunes N. Hanspers K. Willighagen E. L. Bohler A.. (2016). WikiPathways: capturing the full diversity of pathway knowledge. Nucleic Acids Res. 44, D488–D494. 10.1093/nar/gkv1024 26481357 PMC4702772

[B54] Lamarche B. J. Orazio N. I. Weitzman M. D. (2010). The MRN complex in double-strand break repair and telomere maintenance. FEBS Lett. 584, 3682–3695. 10.1016/j.febslet.2010.07.029 20655309 PMC2946096

[B55] Larrea A. A. Lujan S. A. Kunkel T. A. (2010). SnapShot: DNA mismatch repair. Cell 141:730.e1. 10.1016/j.cell.2010.05.002 20478261

[B56] Li D. (2019). “Statistical methods for rna sequencing data analysis,” in Computational Biology, ed H. Husi (Brisbane, QLD: Codon Publications). 31815396

[B57] Li H. Handsaker B. Wysoker A. Fennell T. Ruan J. Homer N.. (2009). The sequence alignment/map format and SAMtools. Bioinformatics 25, 2078–2079. 10.1093/bioinformatics/btp352 19505943 PMC2723002

[B58] Li H. S. Zhou Y. N. Li L. Li S. F. Long D. Chen X. L.. (2019). HIF-1alpha protects against oxidative stress by directly targeting mitochondria. Redox Biol. 25:101109. 10.1016/j.redox.2019.101109 30686776 PMC6859547

[B59] Li Z. Pearlman A. H. Hsieh P. (2016). DNA mismatch repair and the DNA damage response. DNA Repair 38, 94–101. 10.1016/j.dnarep.2015.11.019 26704428 PMC4740233

[B60] Liao Y. Wang J. Jaehnig E. J. Shi Z. Zhang B. (2019). WebGestalt 2019: gene set analysis toolkit with revamped UIs and APIs. Nucleic Acids Res. 47, W199–W205. 10.1093/nar/gkz401 31114916 PMC6602449

[B61] Liu T. Zhang L. Joo D. Sun S. C (2017). NF-kappaB signaling in inflammation. Signal Transduct. Target Ther. 2:17023. 10.1038/sigtrans.2017.23 29158945 PMC5661633

[B62] Lu X. X. Cao L. Y. Chen X. Xiao J. Zou Y. Chen Q. (2016). PTEN inhibits cell proliferation, promotes cell apoptosis, and induces cell cycle arrest via downregulating the PI3K/AKT/hTERT pathway in lung adenocarcinoma A549 cells. Biomed. Res. Int. 2016:2476842. 10.1155/2016/2476842 27822469 PMC5086351

[B63] Ma T. Keller J. A. Yu X. (2011). RNF8-dependent histone ubiquitination during DNA damage response and spermatogenesis. Acta Biochim. Biophys. Sin. 43, 339–345. 10.1093/abbs/gmr016 21444325 PMC3080603

[B64] Marechal A. Zou L. (2013). DNA damage sensing by the ATM and ATR kinases. Cold Spring Harb. Perspect. Biol. 5:a012716. 10.1101/cshperspect.a012716 24003211 PMC3753707

[B65] Marioni J. C. Mason C. E. Mane S. M. Stephens M. Gilad Y. (2008). RNA-seq: an assessment of technical reproducibility and comparison with gene expression arrays. Genome Res. 18, 1509–1517. 10.1101/gr.079558.108 18550803 PMC2527709

[B66] Marston E. Weston V. Jesson J. Maina E. McConville C. Agathanggelou A.. (2009). Stratification of pediatric ALL by *in vitro* cellular responses to DNA double-strand breaks provides insight into the molecular mechanisms underlying clinical response. Blood 113, 117–126. 10.1182/blood-2008-03-142950 18941120

[B67] Mavragani I. V. Nikitaki Z. Kalospyros S. A. Georgakilas A. G. (2019). Ionizing radiation and complex DNA damage: from prediction to detection challenges and biological significance. Cancers 11:1789. 10.3390/cancers11111789 31739493 PMC6895987

[B68] Mavragani I. V. Nikitaki Z. Souli M. P. Aziz A. Nowsheen S. Aziz K.. (2017). Complex DNA damage: a route to radiation-induced genomic instability and carcinogenesis. Cancers 9:91. 10.3390/cancers9070091 28718816 PMC5532627

[B69] Metzker M. L. (2010). Sequencing technologies - the next generation. Nat. Rev. Genet. 11, 31–46. 10.1038/nrg2626 19997069

[B70] Mikkelsen R. B. Wardman P. (2003). Biological chemistry of reactive oxygen and nitrogen and radiation-induced signal transduction mechanisms. Oncogene 22, 5734–5754. 10.1038/sj.onc.1206663 12947383

[B71] Modrich P. (1997). Strand-specific mismatch repair in mammalian cells. J. Biol. Chem. 272, 24727–24730. 10.1074/jbc.272.40.24727 9312062

[B72] Multhoff G. Radons J. (2012). Radiation, inflammation, and immune responses in cancer. Front. Oncol. 2:58. 10.3389/fonc.2012.00058 22675673 PMC3366472

[B73] Nakano K. Vousden K. H. (2001). PUMA, a novel proapoptotic gene, is induced by p53. Mol. Cell 7, 683–694. 10.1016/S1097-2765(01)00214-3 11463392

[B74] Nikitaki Z. Mavragani I. V. Laskaratou D. A. Gika V. Moskvin V. P. Theofilatos K.. (2016). Systemic mechanisms and effects of ionizing radiation: a new “old” paradigm of how the bystanders and distant can become the players. Semin. Cancer Biol. 37–38, 77–95. 10.1016/j.semcancer.2016.02.002 26873647

[B75] Nikitaki Z. Michalopoulos I. Georgakilas A. G. (2015). Molecular inhibitors of DNA repair: searching for the ultimate tumor killing weapon. Future Med. Chem. 7, 1543–1558. 10.4155/fmc.15.95 26306465

[B76] Oda E. Ohki R. Murasawa H. Nemoto J. Shibue T. Yamashita T.. (2000). Noxa, a BH3-only member of the Bcl-2 family and candidate mediator of p53-induced apoptosis. Science 288, 1053–1058. 10.1126/science.288.5468.1053 10807576

[B77] Patel M. Nowsheen S. Maraboyina S. Xia F. (2020). The role of poly(ADP-ribose) polymerase inhibitors in the treatment of cancer and methods to overcome resistance: a review. Cell Biosci. 10:35. 10.1186/s13578-020-00390-7 32180937 PMC7065339

[B78] Patil M. Pabla N. Dong Z. (2013). Checkpoint kinase 1 in DNA damage response and cell cycle regulation. Cell. Mol. Life Sci. 70, 4009–4021. 10.1007/s00018-013-1307-3 23508805 PMC3731415

[B79] Pavlopoulou A. Bagos P. G. Koutsandrea V. Georgakilas A. G. (2017). Molecular determinants of radiosensitivity in normal and tumor tissue. A bioinformatic approach. Cancer Lett. 403, 37–47. 10.1016/j.canlet.2017.05.023 28619524

[B80] Pavlopoulou A. Oktay Y. Vougas K. Louka M. Vorgias C. E. Georgakilas A. G. (2016). Determinants of resistance to chemotherapy and ionizing radiation in breast cancer stem cells. Cancer Lett. 380, 485–493. 10.1016/j.canlet.2016.07.018 27450721

[B81] Pawlowski J. Kraft A. S. (2000). Bax-induced apoptotic cell death. Proc. Natl. Acad. Sci. U. S. A. 97, 529–531. 10.1073/pnas.97.2.529 10639111 PMC33959

[B82] Pertea M. Pertea G. M. Antonescu C. M. Chang T. C. Mendell J. T. Salzberg S. L. (2015). StringTie enables improved reconstruction of a transcriptome from RNA-seq reads. Nat. Biotechnol. 33, 290–295. 10.1038/nbt.3122 25690850 PMC4643835

[B83] Pezuk J. A. Brassesco M. S. Morales A. G. de Oliveira J. C. de Oliveira H. F. Scrideli C. A.. (2013). Inhibition of polo-like kinase 1 induces cell cycle arrest and sensitizes glioblastoma cells to ionizing radiation. Cancer Biother. Radiopharm. 28, 516–522. 10.1089/cbr.2012.1415 23713868 PMC3741430

[B84] Porta C. Paglino C. Mosca A. (2014). Targeting PI3K/Akt/mTOR signaling in cancer. Front. Oncol. 4:64. 10.3389/fonc.2014.00064 24782981 PMC3995050

[B85] Porter A. G. Janicke R. U. (1999). Emerging roles of caspase-3 in apoptosis. Cell Death Differ. 6, 99–104. 10.1038/sj.cdd.4400476 10200555

[B86] Pouyssegur J. Dayan F. Mazure N. M. (2006). Hypoxia signalling in cancer and approaches to enforce tumour regression. Nature 441, 437–443. 10.1038/nature04871 16724055

[B87] Ramasamy A. Mondry A. Holmes C. C. Altman D. G. (2008). Key issues in conducting a meta-analysis of gene expression microarray datasets. PLoS Med. 5:e184. 10.1371/journal.pmed.0050184 18767902 PMC2528050

[B88] Robinson M. D. McCarthy D. J. Smyth K. G. (2010). edgeR: a bioconductor package for differential expression analysis of digital gene expression data. Bioinformatics 26, 139–140. 10.1093/bioinformatics/btp616 19910308 PMC2796818

[B89] Robinson M. D. Oshlack A. (2010). A scaling normalization method for differential expression analysis of RNA-seq data. Genome Biol. 11:R25. 10.1186/gb-2010-11-3-r25 20196867 PMC2864565

[B90] Rouse J. Jackson S. P. (2002). Interfaces between the detection, signaling, and repair of DNA damage. Science 297, 547–551. 10.1126/science.1074740 12142523

[B91] Sakellaropoulos T. Vougas K. Narang S. Koinis F. Kotsinas A. Polyzos A.. (2019). A deep learning framework for predicting response to therapy in cancer. Cell Rep. 29, 3367–3373.e4. 10.1016/j.celrep.2019.11.017 31825821

[B92] Sayers E. W. Beck J. Brister J. R. Bolton E. E. Canese K. Comeau D. C.. (2020). Database resources of the national center for biotechnology information. Nucleic Acids Res. 48, D9–D16. 10.1093/nar/gkz899 31602479 PMC6943063

[B93] Schoenhals J. E. Seyedin S. N. Tang C. Cortez M. A. Niknam S. Tsouko E.. (2016). Preclinical rationale and clinical considerations for radiotherapy plus immunotherapy: going beyond local control. Cancer J. 22, 130–137. 10.1097/PPO.0000000000000181 27111909

[B94] Smith D. Mann D. Yong K. (2016). Cyclin D type does not influence cell cycle response to DNA damage caused by ionizing radiation in multiple myeloma tumours. Br. J. Haematol. 173, 693–704. 10.1111/bjh.13982 27146121

[B95] Sonenshein G. E. (1997). Rel/NF-kappa B transcription factors and the control of apoptosis. Semin. Cancer Biol. 8, 113–119. 10.1006/scbi.1997.0062 9299589

[B96] Soubeyrand S. Pope L. Pakuts B. Hache R. J. (2003). Threonines 2638/2647 in DNA-PK are essential for cellular resistance to ionizing radiation. Cancer Res. 63, 1198–1201. 12649176

[B97] Spratt D. E. Speers C. (2019). RadioGx: a new preclinical tool to model intrinsic radiosensitivity. Cancer Res. 79, 6076–6078. 10.1158/0008-5472.CAN-19-3277 31836601

[B98] StataCorp (2013). Stata Statistical Software: Release 13. College Station, TX: StataCorpLP.

[B99] Stylianou S. Clarke R. B. Brennan K. (2006). Aberrant activation of notch signaling in human breast cancer. Cancer Res. 66, 1517–1525. 10.1158/0008-5472.CAN-05-3054 16452208

[B100] Sulai N. H. Tan A. R. (2018). Development of poly(ADP-ribose) polymerase inhibitors in the treatment of BRCA-mutated breast cancer. Clin. Adv. Hematol. Oncol. 16, 491–501. 30067621

[B101] Sutherland B. M. Bennett P. V. Sidorkina O. Laval J. (2000). Clustered DNA damages induced in isolated DNA and in human cells by low doses of ionizing radiation. Proc. Natl. Acad. Sci. U. S. A. 97, 103–108. 10.1073/pnas.97.1.103 10618378 PMC26623

[B102] Swanton E. Savory P. Cosulich S. Clarke P. Woodman P. (1999). Bcl-2 regulates a caspase-3/caspase-2 apoptotic cascade in cytosolic extracts. Oncogene 18, 1781–1787. 10.1038/sj.onc.1202490 10086332

[B103] Syed V. (2016). TGF-beta signaling in cancer. J. Cell Biochem. 117, 1279–1287. 10.1002/jcb.25496 26774024

[B104] Szklarczyk D. Gable A. L. Lyon D. Junge A. Wyder S. Huerta-Cepas J.. (2019). STRING v11: protein-protein association networks with increased coverage, supporting functional discovery in genome-wide experimental datasets. Nucleic Acids Res. 47, D607–D613. 10.1093/nar/gky1131 30476243 PMC6323986

[B105] Taalman R. D. Jaspers N. G. Scheres J. M. de Wit J. Hustinx T. W. (1983). Hypersensitivity to ionizing radiation, *in vitro*, in a new chromosomal breakage disorder, the Nijmegen breakage syndrome. Mutat. Res. 112, 23–32. 10.1016/0167-8817(83)90021-4 6828038

[B106] Tang C. Wang X. Soh H. Seyedin S. Cortez M. A. Krishnan S.. (2014). Combining radiation and immunotherapy: a new systemic therapy for solid tumors? Cancer Immunol. Res. 2, 831–838. 10.1158/2326-6066.CIR-14-0069 25187273 PMC5367158

[B107] Tang Z. Li C. Kang B. Gao G. Li C. Zhang Z. (2017). GEPIA: a web server for cancer and normal gene expression profiling and interactive analyses. Nucleic Acids Res. 45, W98–W102. 10.1093/nar/gkx247 28407145 PMC5570223

[B108] Unger K. (2014). Integrative radiation systems biology. Radiat. Oncol. 9:21. 10.1186/1748-717X-9-21 24411063 PMC3901372

[B109] Van den Bossche J. Domen A. Peeters M. Deben C. De Pauw I. Jacobs J.. (2019). Radiosensitization of non-small cell lung cancer cells by the Plk1 inhibitor volasertib is dependent on the p53 status. Cancers 11:1893. 10.3390/cancers11121893 31795121 PMC6966428

[B110] Vougas K. Sakellaropoulos T. Kotsinas A. Foukas G. P. Ntargaras A. Koinis F.. (2019). Machine learning and data mining frameworks for predicting drug response in cancer: an overview and a novel *in silico* screening process based on association rule mining. Pharmacol. Ther. 203:107395. 10.1016/j.pharmthera.2019.107395 31374225

[B111] Wang M. Chen X. Chen H. Zhang X. Li J. Gong H.. (2015). RNF8 plays an important role in the radioresistance of human nasopharyngeal cancer cells *in vitro*. Oncol. Rep. 34, 341–349. 10.3892/or.2015.3958 25955491

[B112] Wang W. Yang L. Hu L. Li F. Ren L. Yu H.. (2013). Inhibition of UBE2D3 expression attenuates radiosensitivity of MCF-7 human breast cancer cells by increasing hTERT expression and activity. PLoS ONE 8:e64660. 10.1371/journal.pone.0064660 23741361 PMC3669415

[B113] Wang Y. Branicky R. Noe A. Hekimi S. (2018). Superoxide dismutases: dual roles in controlling ROS damage and regulating ROS signaling. J. Cell Biol. 217, 1915–1928. 10.1083/jcb.201708007 29669742 PMC5987716

[B114] Weng L. Brown J. Eng C. (2001). PTEN induces apoptosis and cell cycle arrest through phosphoinositol-3-kinase/Akt-dependent and -independent pathways. Hum. Mol. Genet. 10, 237–242. 10.1093/hmg/10.3.237 11159942

[B115] Yamamori T. Yasui H. Yamazumi M. Wada Y. Nakamura Y. Nakamura H.. (2012). Ionizing radiation induces mitochondrial reactive oxygen species production accompanied by upregulation of mitochondrial electron transport chain function and mitochondrial content under control of the cell cycle checkpoint. Free Radic. Biol. Med. 53, 260–270. 10.1016/j.freeradbiomed.2012.04.033 22580337

[B116] Yang J. Xu Z. P. Huang Y. Hamrick H. E. Duerksen-Hughes P. J. Yu Y. N. (2004). ATM and ATR: sensing DNA damage. World J. Gastroenterol. 10, 155–160. 10.3748/wjg.v10.i2.155 14716813 PMC4716994

[B117] Zhang B. Kirov S. Snoddy J. (2005). WebGestalt: an integrated system for exploring gene sets in various biological contexts. Nucleic Acids Res. 33, W741–W748. 10.1093/nar/gki475 15980575 PMC1160236

[B118] Zhao S. Weng Y. C. Yuan S. S. Lin Y. T. Hsu H. C. Lin S. C.. (2000). Functional link between ataxia-telangiectasia and Nijmegen breakage syndrome gene products. Nature 405, 473–477. 10.1038/35013083 10839544

