# Supplementary figures and images for: Investigating Molecular Determinants of Cancer Cell Resistance to Ionizing Radiation Through an Integrative Bioinformatics Approach
Source: Front Cell Dev Biol. 2021 Apr 7;9:620248. doi: 10.3389/fcell.2021.620248 (PMC8058375; doi:10.3389/fcell.2021.620248)

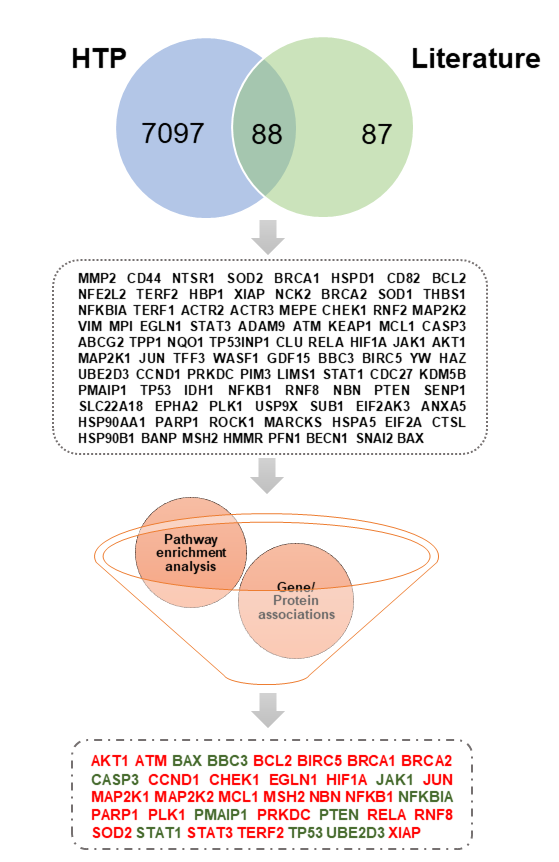

Supplement: Supplementary Figure 1 — Schematic workflow outlining the selection of radiogenes. Venn diagram comparing the high-throughput (HTP) and literature-derived cancer radioresistance-associated genes. Functional enrichment and interaction network analysis of the common 88 genes resulted in 36 genes. Red, up-regulated genes; Green, down-regulated genes. [file Image_1.TIF]

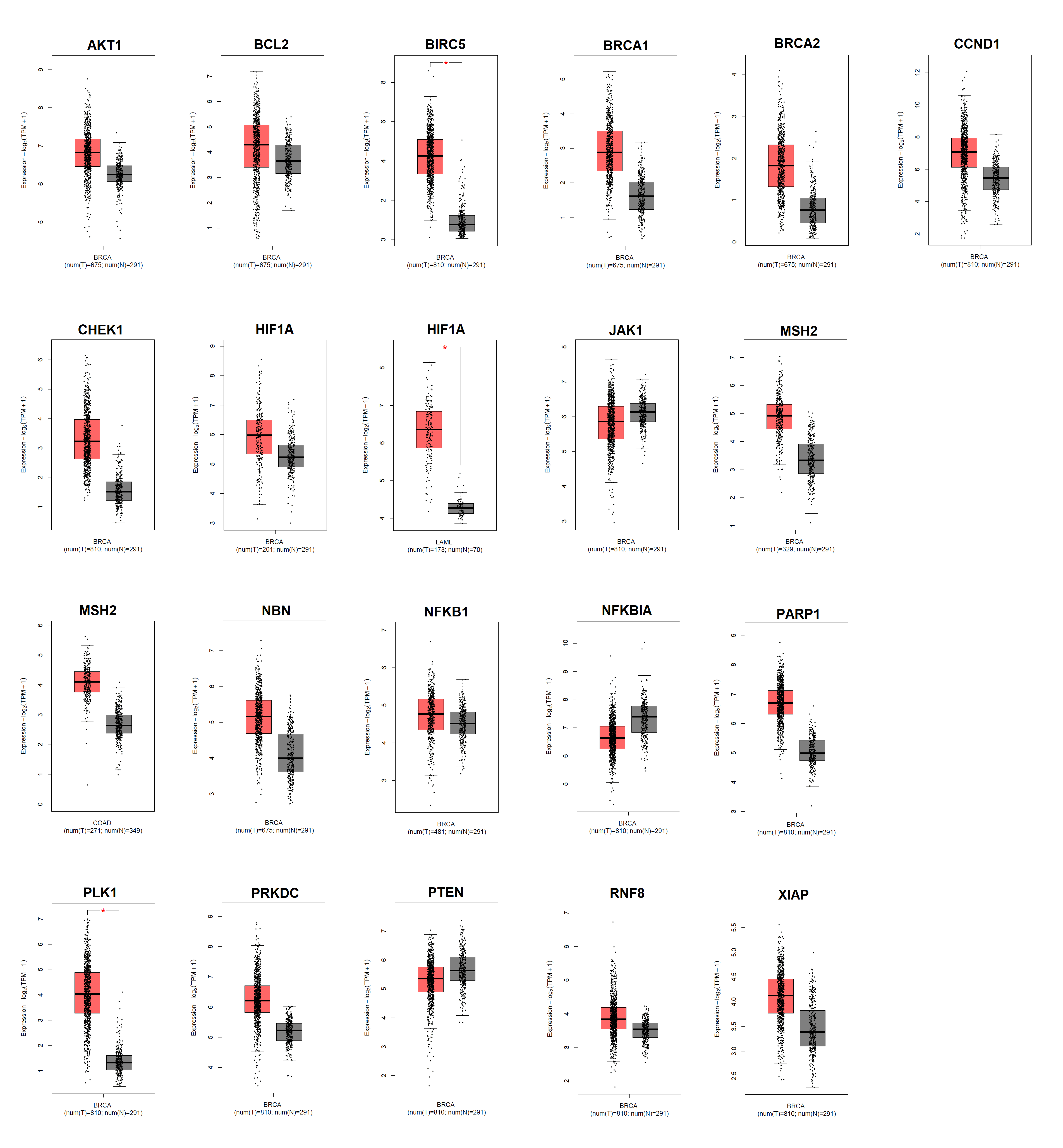

Supplement: Supplementary Figure 2 — Differential expression patterns of radiogenes in the corresponding cancer tissue; BRCA, breast invasive carcinoma; COAD, colon adenocarcinoma; LAML, acute myeloid leukemia. The red box represents cancer tissue samples and the gray box indicates normal samples. [file Image_2.TIF]

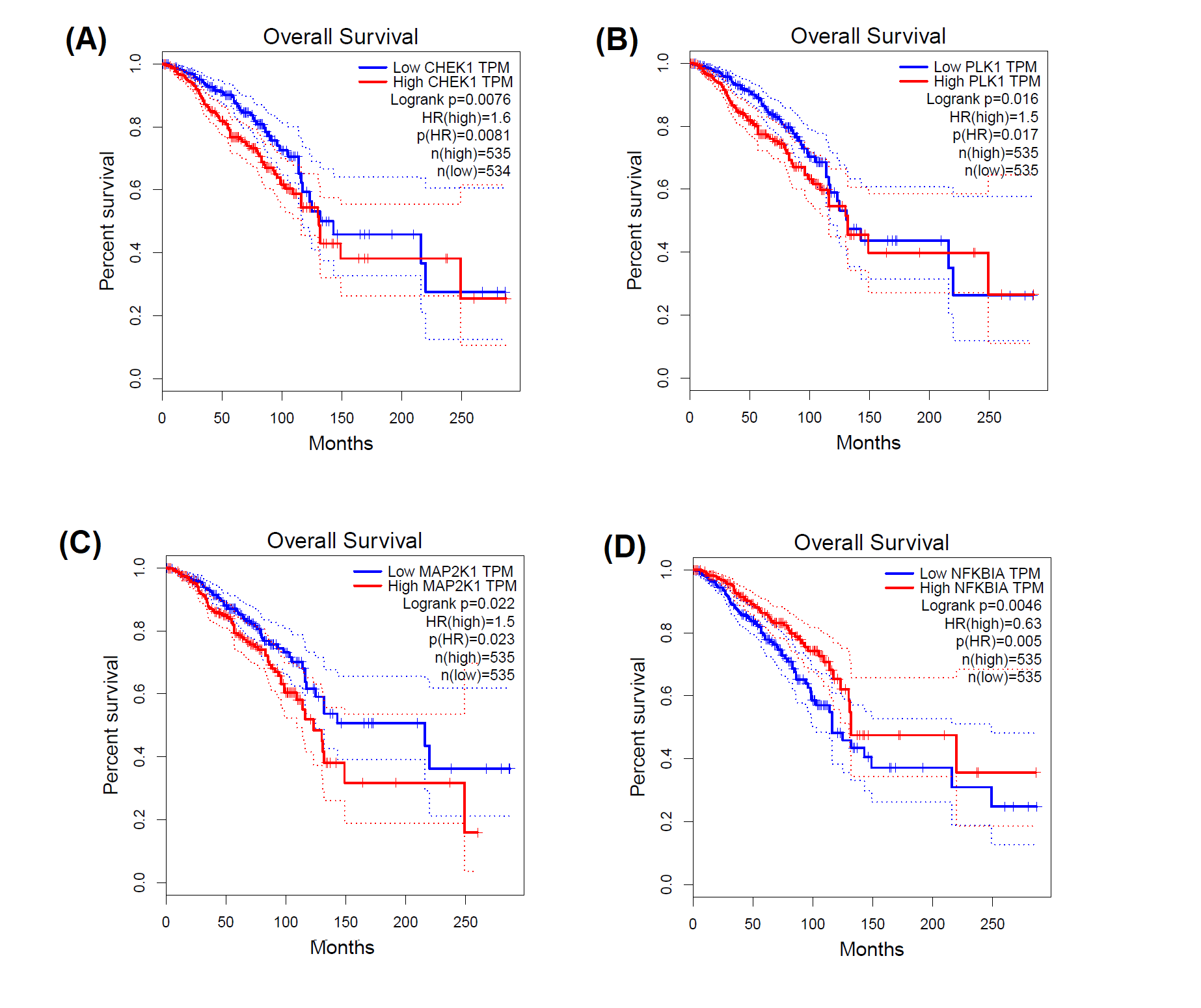

Supplement: Supplementary Figure 3 — Kaplan-Meier curves depicting the prognostic value of radiogenes for overall survival in invasive breast carcinoma (A) CHEK1, (B) PLK1, (C) MAP2K1 and (D) NFKBIA. The HR “HR(high)” and the corresponding p-values “p(HR)” are shown. The 95% confidence intervals (CI) are denoted by dotted lines. The number of high-risk and low-risk patients are indicated by “n(high)” and “n(low),” respectively. [file Image_3.TIF]
